# Suspected adverse drug reactions of the type 2 antidiabetic drug class dipeptidyl‐peptidase IV inhibitors (DPP4i): Can polypharmacology help explain?

**DOI:** 10.1002/prp2.1029

**Published:** 2022-12-05

**Authors:** Lauren Jones, Alan M. Jones

**Affiliations:** ^1^ Medicines Safety Research Group (MSRG), School of Pharmacy University of Birmingham Birmingham UK

**Keywords:** dipeptidyl‐peptidase IV inhibitors, drug‐related side effects, adverse drug reactions, pharmacokinetics, polypharmacology

## Abstract

To interpret the relationship between the polypharmacology of dipeptidyl‐peptidase IV inhibitors (DPP4i) and their suspected adverse drug reaction (ADR) profiles using a national registry. A retrospective investigation into the suspected ADR profile of four licensed DPP4i in the United Kingdom using the National MHRA Yellow Card Scheme and OpenPrescribing databases. Experimental data from the ChEMBL database alongside physiochemical (PC) and pharmacokinetic (PK) profiles were extracted and interpreted. DPP4i show limited polypharmacology alongside low suspected ADR rates. We found a minimal statistical difference between the unique ADR profiles ascribed to the DPP4i except for total ADRs (*χ* ^2^; *p* < .05). Alogliptin consistently showed the highest suspected ADR rate per 1 000 000 items prescribed. Saxagliptin showed the lowest suspected ADR rate across all organ classes but did not reach statistical difference (*χ* ^2^; *p* > .05). We confirmed the Phase III clinical trial data that showed gastrointestinal and skin reactions are the most reported ADRs across the DPP4i class and postulated underlying mechanisms for this based on possible drug interactions. The main pharmacological mechanism behind the ADRs is attributed to interactions with DPP4 activity and/or structure homolog (DASH) proteins which augment the immune‐inflammatory modulation of DPP4.

## Graphical abstract

Role of polypharmacology in suspected dipeptidyl‐peptidase IV inhibitors adverse drug reactions.

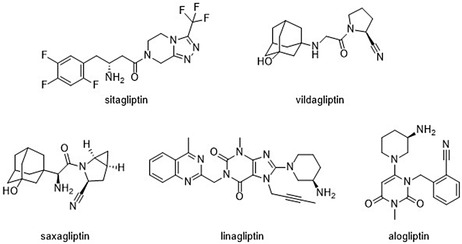

**Abbreviations**
ADME: absorption, distribution, metabolism, and excretion
ADR: adverse drug reaction
BBB: blood‐brain barrier
BP: bullous pemphigoid
ChEMBL: chemical database of bioactive molecules of the European Molecular Biology Laboratory
*C*_max_
: peak plasma concentration
CYP: cytochrome P450
DASH: database of aligned structural homologs
DKA: diabetic ketoacidosis
DPP: dipeptidyl‐peptidase
EMA: European Medicines Agency
FAP: fibroblast activation protein
FDA: Food and Drug Administration
GIP: glucose‐dependent insulinotropic polypeptide
GLP‐1: glucagon‐like peptide‐1
HBA: hydrogen bond acceptor
HBD: hydrogen bond donor
HCP: healthcare professional
HF: heart failure
iDAP: interactive drug analysis profiles
LLE: ligand lipophilicity efficiency
M1: muscarinic acetylcholine receptor 1
MCP‐1: monocyte chemoattractant protein‐1
MHRA: medicines and healthcare products regulatory agency
NHS: National Health Service
PC: physicochemical
P‐gp: P‐glycoprotein
PK: pharmacokinetic
PPB: plasma protein binding
SGLT2i: sodium‐glucose co‐transporter 2 inhibitor
SPC: summary of product characteristics
SU: sulfonylurea
^*t*^PSA: total polar surface area
UK: United Kingdom
Vd: volume of distribution

## 1 INTRODUCTION

Type II diabetes is a metabolic disorder primarily associated with insulin sensitivity causing a functional deficit (insulin resistance) which may deteriorate into reduced excretion. Diabetes mellitus is the 9th leading cause of death worldwide^1^ and often associated with cardiovascular events such as myocardial infarction or stroke. In the United Kingdom (UK), diabetes is estimated to account for a 10% of the National Health Service (NHS) budget.^2^

UK clinical guidance for type II diabetes recommends lifestyle advice and modification as the first‐line therapy, followed by metformin as the second‐line therapy.^2^ Intensification of treatment comprises “add‐on” therapies (five are licensed in the UK); Dipeptidyl peptidase‐4 inhibitors (DPP4i), pioglitazone, sulfonylureas (SU), sodium‐glucose co‐transporter 2 inhibitors (SGLT2i) and, glucagon‐like peptide‐1 (GLP‐1) analogs, which can only be initiated under specialist care.

DPP4 is an enzyme that degrades the incretin hormones GLP‐1 and glucose‐dependent insulinotropic polypeptide (GIP), which serves to control glycaemic levels post‐prandially by stimulating insulin synthesis and secretion from pancreatic β‐cells and reducing glucagon secretion from α‐cells.^3^, ^4^, ^5^ Additionally, GIP/GLP‐1 delays gastric emptying and exert central nervous system modulation, thereby increasing satiety and reducing further food intake. DPP4i increases the circulating incretin hormones levels for longer post‐prandially, leading to better glycaemic control, and has shown preservation of pancreatic β‐cell function through increased cell stimulation, proliferation, differentiation, and survival.^6^, ^7^, ^8^ Theoretically, this could slow, or reverse disease progression.

DPP4is have preferential effects over other drug classes depending on patient factors. For example, DPP4i's are considered weight neutral, having a negligible risk of hypoglycemia,^4^, ^7^ whereas SU and pioglitazone have a higher risk of hypoglycaemic episodes and are associated with weight gain.^9^, ^10^, ^11^ Pioglitazone cannot be initiated in patients with heart failure (HF) due to potential complications.^10^ SGLT2i's are associated with a higher risk of diabetic ketoacidosis (DKA) and limb and foot amputations.^12^ GLP‐1 analogs have poor oral bioavailability and are administered via injection.

Adverse drug reactions (ADR) are the unintended side effects of drugs at clinical doses for an indicated disease. ADRs can significantly impact the pharmaceutical management of up to 20% of hospitalized patients' and 25% of outpatient's care.^13^ This is associated with a significant cost; associated NHS hospitalizations equate to £380 M per year.^14^

Polypharmacology is how a single drug interacts with multiple targets.^15^ Side effects can be a result of interactions with both the desired target and/or other targets within the body, potentially causing harm or death.^13^

**FIGURE 1:**
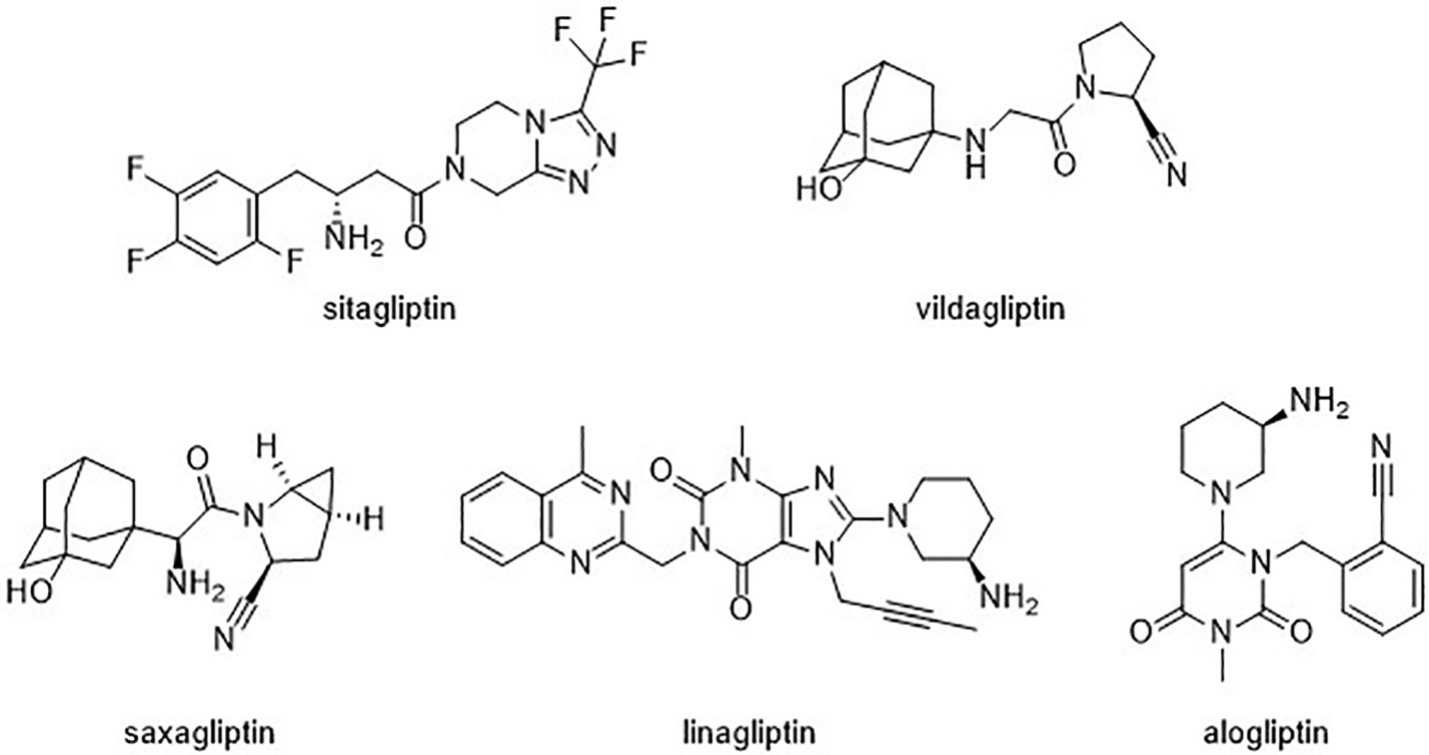
Chemical structures of the DPP4i studied.

The UK's Medicines and Healthcare products Regulatory Agency (MHRA) Yellow Card Scheme^16^ involves voluntary reporting from healthcare professionals and patients. It provides post‐marketing surveillance of observational ADRs of UK‐licensed drugs in complex real‐world settings.^17^ Monitoring with this spontaneous reporting system identifies further unanticipated or previously undetected ADRs. This information influences future clinical guidance. At the time of writing, there are five UK‐licensed DPP4i (approval date in parentheses): sitagliptin (2007); vildagliptin (2007), saxagliptin (2009), linagliptin (2011), and alogliptin (2013) (Figure 1).

Research linking polypharmacology and suspected ADR profiles of DPP4i have to the best of our knowledge not been conducted. Investigation of the polypharmacology of each DPP4i and attributing polypharmacology to ADRs could potentially enable prospective ADR prediction in this drug class or indeed others.^18^, ^19^ Furthermore, where an ADR profile is significantly harmful, which may be attributed to its polypharmacology, this may influence clinical guidance or associated clinical decision making.

The aims of this study are to:

- Determine the unique polypharmacology of each DPP4i class member by assessing the affinity of each drug to a series of tested physiological enzymes.
- Use a national registry (Yellow Card Scheme) to determine suspected ADR signal rates relative to prescribing levels in the population.
- Run statistical analyses to ascertain which ADR signals are significant.
- Attempt to establish links between each DPP4i's unique polypharmacology and suspected ADR profile by postulating underlying mechanisms.

The objective of this research is to ascertain the links (if any) between the polypharmacology of a DPP4i and the reported ADR profile, relative to prescribing levels.

## 2 METHODS

### 2.1 Physiochemical properties

Physiochemical (PC) properties influence the absorption, distribution, metabolism, and excretion (ADME) of a given drug and are key to determine the activity in vivo. These properties were compared across the DPP4i class to determine whether they influenced the pharmacology and side effect profile of each drug. Physiochemical properties such as p*K*_*a*_ (‐log_10_ *K*_*a*_; where *K*_*a*_ is the acid dissociation constant), hydrogen bond donators/acceptors (HBDs/HBAs), Log_10_P (experimentally measured partition coefficient of a substance between organic and aqueous environments in its neutral form) and cLog_10_P (calculated partition coefficient) were data‐mined from the Chemical database of bioactive molecules of the European Molecular Biology Laboratory (ChEMBL), Wellcome Trust Genome Campus, Hinxton, UK^20^ and molecular weight and topological polar surface area (^*t*^PSA) from PubChem.^21^ Log_10_D^7.4^ (Distribution coefficient is the partition coefficient for an ionized compound) was calculated from the respective Log_10_P, p*K*_*a*_, and pH values. Log_10_D^7.4^ gives an indication of the distribution between the aqueous and organic phases at pH = 7.4 and indicates the lipophilicity of the molecule in an ionized state. ^*t*^PSA considers the polar interactions on the surface of the molecule in deciding the conformational shape of a drug and is linked to oral absorption and blood–brain barrier (BBB) penetration.^22^

### 2.2 Pharmacokinetic properties

Pharmacokinetic (PK) properties were characterized by the European Medicines Agency (EMA) Summary of Product Characteristics (SPC) profiles.^23^, ^24^, ^25^, ^26^ PK properties included *C*_max_ (peak plasma concentration), volume of distribution (V_d_), plasma protein binding (PPB), half‐life (*t*_1/2_), renal clearance (Cl), bioavailability (%*F*), and method of clearance/excretion. Where the *C*_max_ was reported in alternative units (ng/mL), it was converted to nM to compare against target IC_50_ values (nM) to determine if the reported value had a physiological relevance.

*p*IC_50_ was calculated as the ‐Log_10_(IC_50_) from the median IC_50_ value of the intended target, DPP4, and used to determine the ligand‐lipophilicity efficiency (LLE = *p*IC_50_ – clog_10_P) which indicates the promiscuity of the molecule from the target enzyme. LLE quantifies potency with a target against lipophilicity and a lower value is associated with more toxicity (<5 is considered toxic).^27^ Where parameters were not given in the resources listed, literature searches using SciFinder® for the PK property + drug name were used to gather missing information.^28^, ^29^, ^30^

### 2.3 Blood–Brain Barrier (BBB) penetration

The threshold of BBB penetration was assigned using; molecular weight < 450 Da; neutral or basic drugs (determined by p*K*_a_); ^*t*^PSA < 90 Å; <6 hydrogen bond donors; <2 hydrogen bond acceptors; log_10_D^7.4^ 1–3; and whether it is a P‐glycoprotein (P‐gp) substrate. The more BBB penetrant properties a drug possesses, the more likely it will cross the BBB.^31^

### 2.4 Pharmacological properties

Pharmacological target searches were completed using drug name searches on ChEMBL (accessed 29/10/2021)^20^; a database that extracts and standardizes bioactivity data from medicinal chemistry journals and other databases to identify biological interactions.^32^, ^33^, ^34^ Median IC_50_ values were calculated to negate extremities of values, as IC_50_ values were tested across multiple labs at different time points. IC_50_ quantitively shows the concentration of a substance required to inhibit a protein by 50%. ChEMBL searches were refined to single protein assays (*homosapien*) to make the physiological activity as relevant as possible for human use. Those with interactions >100 000 nM were disregarded from reporting due to insignificant physiological relevance.

### 2.5 Prescribing data

The Openprescribing.net resource provided prescribing data in primary care across England from January 2017 to August 2021.^35^ The prescription items (*R*_*x*_) were collated to standardize the suspected ADRs and to consider differing prescribing rates between drugs within the class. This is different from typical pharmacovigilance studies (that do not consider drug prescribing levels) and enables a surrogate standardization for suspected ADRs to be more readily compared between drug class members.

### 2.6 Adverse drug reactions (ADR) data

ADR data were collated from the MHRA Yellow Card Scheme Interactive Drug Analysis Profiles (iDAP)^16^ with dates set between January 2017 to August 2021 for single active constituents only. The categories reported were system organ classes; fatalities; sub‐category terms selected by a threshold ADR rate of 1.5 ADRs/1000000 *R*_*x*_ for at least one of the four drugs. The Yellow Card Scheme did not report ADRs for vildagliptin after December 2020 and, therefore, did not fit the scope of this research, which considered ADRs between January 2017 to August 2021. Vildagliptin was therefore excluded from further analysis due to incomplete datasets.

### 2.7 Statistical analysis

Chi‐squared (χ^2^) analysis was performed on the standardized ADR/1000000 *R*_*x*_ using Microsoft Excel Version 16.55. The test was performed across all four drugs (Table 2) and then drug vs drug analysis (S1). A *p*‐value of <.05 was set for statistical significance.

## 3 RESULTS

### 3.1 Physiochemical property results

Chemical properties including molecular weight, ^*t*^PSA and LLE were similar across the class of DPP4i (Table S2). All DPP4i had LLE >5 suggesting they are non‐promiscuous inhibitors, with saxagliptin (8.82) possessing the most efficient binding and linagliptin (6.20) the least. All DPP4i, except for linagliptin, had a negative Log_10_D^7.4^, which is associated with hydrophilicity. Saxagliptin exhibited the highest potential to cross the BBB and therefore central nervous system effects may be anticipated. The remaining DPP4i studied had a low propensity to cross the BBB.

### 3.2 Pharmacokinetic results

All reported drugs have once‐daily administration at varied doses and have similar half‐lives. Notably, linagliptin had a high *V*_d_ which suggests a higher distribution around tissues in the body, and this may be explained by the Log_10_D^7.4^ value being the most lipophilic of the DPP4i studied. Furthermore, linagliptin had the lowest oral bioavailability, significantly higher PPB, and reduced renal clearance.

### 3.3 Pharmacological properties

Linagliptin had the strongest interaction with DPP4 (1.0 nM), followed by saxagliptin (3.4 nM) and alogliptin (5.3 nM). Sitagliptin showed the weakest activity with DPP4 (18 nM). Linagliptin also showed a stronger affinity for Fibroblast Activation Protein (FAP) (89 nM) as well as activity with M1 receptors (Table 1). Saxagliptin had an inhibitory activity with FAP, but stronger affinity interactions with proteins DPP8 and DPP9. However, these interactions should not be considered in isolation; given that the *C*_max_ is considerably lower than off‐target interactions, they may not be physiologically relevant. Key interactions are shown in Table 1.

**TABLE 1:** Summarized median IC_50_ values (nM) for four DPP4i against named proteins

	Alogliptin	Linagliptin	Saxagliptin	Sitagliptin
DPP4	5.3	1	3.4	18
DPP8	100 000	70 000	242	48 000
DPP9	100 000	100 000	102	100 000
FAP	100 000	89	1000	100 000
M1		297.5		
*C*_max_ (nM)	295.8	11–12	68.2	950

	1–10 nM			
	10–100 nM			
	100–1000 nM			
	1000–10 000 nM			
	Weak inhibition (>10 000 nM)			
	Undetermined			

*Note*: For a full list of interactions see S3. The C_max_ (nM) of each DPP4i is provided to compare physiological relevance. Only homosapien, single protein assays were included in the averaged data.^20^ Key shows color which correlates to the stated strength interaction. Proteins: DPP, Dipeptidyl peptidase; FAP, Fibroblast Activation Protein; M1, Muscarinic acetylcholine receptor 1; CYP, Cytochrome P450. C_max_—peak plasma concentration.

### 3.4 Open prescribing

Prescribing data shows that sitagliptin is the most prescribed DPP4i, with 11.5 million items (*R*_*x*_), followed by linagliptin (9.4 million *R*_*x*_), alogliptin (5.6 million *R*_*x*_), and saxagliptin (1.0 million *R*_*x*_). Number of prescriptions does not necessarily equate to the number of patients as the drugs are available in a variety of formulated tablet strengths (S4) and multiple tablets may be required to reach the daily indicated dose.

### 3.5 ADR results

Given the significant difference in prescribed numbers between the four drugs, the ADRs were standardized per 1 000 000 *R*_*x*_ for accurate comparison whilst mitigating the risk of misinterpreting unstandardized values.

### 3.6 Total ADRs

Alogliptin had the highest total ADR rate with 98.18 per 1 000 000 Rx followed by linagliptin (69.81), sitagliptin (55.24), and saxagliptin (53.01) (Table 2). This result was statistically significant across the DPP4i class (Table 2) with a *p* < .05. Furthermore, alogliptin reached statistical difference when analyzed individually against other DPP4i studied (S1).

**TABLE 2:** Adverse Drug Reactions for the DPP4i studied

	Alogliptin	Linagliptin	Saxagliptin	Sitagliptin	*p* value
Total *Rx*	5 611 886	9 425 563	1 056 504	11 459 570	
Fatalities	1 (0.18)	4 (0.42)	0 (0)	6 (0.52)	>.05
Cardiac disorders	16 (2.85)	9 (0.95)	1 (0.95)	4 (0.35)	>.05
Eye disorders	21 (3.74)	24 (2.55)	2 (1.89)	3 (0.26)	>.05
Gastrointestinal disorders	156 (27.80)	178 (18.88)	13 (12.30)	172 (15.01)	>.05
Acute and chronic pancreatitis	10 (1.78)	25 (2.65)	2 (1.89)	30 (2.62)	>.05
Diarrhea	18 (3.21)	21 (2.23)	1 (0.95)	22 (1.92)	>.05
Gastrointestinal and abdominal pains	33 (5.88)	28 (2.97)	3 (2.84)	33 (2.88)	>.05
Nausea and vomiting symptoms	41 (7.31)	26 (2.76)	2 (1.89)	26 (2.27)	>.05
General disorders and administration site conditions	60 (10.69)	57 (6.05)	6 (5.68)	73 (6.37)	>.05
Asthenic conditions	23 (4.10)	15 (1.59)	2 (1.89)	13 (1.13)	>.05
Infections	7 (1.25)	13 (1.38)	4(3.79)	11(0.96)	>.05
Metabolism and nutrition disorders	15 (2.67)	10 (1.06)	3 (2.84)	23 (2.01)	>.05
Musculoskeletal and connective tissue disorders	35 (6.24)	42 (4.46)	2 (1.89)	44 (3.84)	>.05
Joint related signs and symptoms	19 (3.39)	17 (1.80)	1 (0.95)	13 (1.13)	>.05
Nervous system disorders	52 (9.27)	65 (6.90)	2 (1.89)	82 (7.16)	>.05
Headaches	14 (2.49)	13 (1.38)	0 (0.00)	15 (1.31)	>.05
Psychiatric disorders	27 (4.81)	28 (2.97)	5 (4.73)	21 (1.83)	>.05
Respiratory, thoracic, and mediastinal disorders	22 (3.92)	43 (4.56)	1 (0.95)	25 (2.18)	>.05
Dyspnoea	8 (1.43)	15 (1.59)	0 (0.00)	5 (0.44)	>.05
Skin and subcutaneous tissue disorders	107 (19.07)	125 (13.26)	5 (4.73)	65 (5.67)	.005
Bullous conditions	9 (1.60)	38 (4.03)	1 (0.95)	13 (1.13)	> .05
Pruritus	29 (5.17)	19 (2.02)	1 (0.95)	11 (0.96)	>.05
Rashes, eruptions and exanthems	45 (8.02)	34 (3.61)	1 (0.95)	16 (1.40)	.03
Total ADRs	551 (98.18)	658 (69.81)	56 (53.01)	633 (55.24)	.0003

*Note*: Data were collected from January 2017 to August 2021 from the Yellow Card Scheme and standardized using prescribing data from openprescribing.net.^16^, ^35^ Number of ADRs given with ADR/1000000 *R*_*x*_ in parenthesis, given to 2 decimal places. *p* values were determined by *χ* ^*2*^ analysis. Reported by organ class, fatalities and grouped with sub‐categorical ADRs which reached the threshold of 1.5 ADRs/1000000 *R*_*x*_. *R*_*x*_—Prescription items.

### 3.7 ADR summary

Alogliptin had the highest ADR rate in cardiac, eye, gastrointestinal, general, musculoskeletal, and connective tissue, nervous system, psychiatric, and skin disorders. In cardiac ADRs, alogliptin (2.85) showed a 3‐fold increase on the next highest rate (0.95 for linagliptin and saxagliptin, respectively) and an 8‐fold difference over sitagliptin (0.35); however, this did not reach a statistical difference. Alogliptin reached a statistical difference against sitagliptin in skin disorders and saxagliptin in gastrointestinal, nervous, and skin disorders.

Saxagliptin consistently showed the lowest ADR/1000000 *R*_*x*_ rate for most ADR types except for metabolism and nutrition disorders (2.84) and infections (3.79), where it showed the highest rate across the four drugs.

Sitagliptin showed the highest fatality rate (0.52) and saxagliptin the lowest with no recorded fatalities; however, this did not reach statistical difference.

Overall, the highest‐rated ADRs across the class were gastrointestinal and skin disorders. In both organ classes, alogliptin experienced the highest rates (27.80 and 19.07, respectively), followed by linagliptin (18.88 and 13.26), sitagliptin (15.01 and 5.67), and saxagliptin (12.30 and 4.73).

## 4 DISCUSSION

The dipeptidyl‐peptidase (DPP) family of enzymes includes homologous enzymes DPP2, DPP4, DPP8, DPP9, and FAP. All are *N*‐terminal dipeptide cleaving serine proteases with preferential action where the second amino acid is proline or alanine were the most seen multitarget interactions of the DPP4i's studied (Table 1).^4^, ^5^, ^36^ These proteins are known as DPP4 activity and/or structure homolog (DASH) proteins.^37^ All members of the family have physiological processes associated with the immune system and inflammation,^38^ which is attributed to their similar mechanisms of action and similar structures. Whilst DPP4 and FAP are extracellular, DPP8 and DPP9 are intracellular, specifically localized to the nucleus and cytosol,^38^, ^39^ which may make their function more redundant than that of DPP4. The exact physiological functions of DPP8 and DPP9 have yet to be fully elucidated.^37^ DPP4 is both a type II transmembrane protein and has a soluble form that lacks the cytoplasmic tail and transmembrane region which circulates in plasma.^36^ DPP4 is ubiquitously expressed, with particularly high expression in the kidney, lung, liver, and small intestine,^37^ as well as circulating enzymatic activity as the cleaved domain continues to exert the effects in serum.

Notably, the *C*_max_ for all four drugs are too low to reach the necessary concentrations to inhibit off‐target proteins such as DPP8, DPP9, and Fibroblast Activation Protein (FAP). This might explain why the suspected ADR rates across the drug class are not large or statistically different as the DPP4i class have similar pharmacological actions. The only clinically likely IC_50_ relative to the C_max_ would be linagliptin interacting with FAP and M1 and saxagliptin with DPP8 and DPP9, although there is a large discrepancy between the two values. Potential accumulation of the drug might occur through decreased excretion or metabolism or accumulation over multiple doses depending on the half‐life of the drug.^40^

Clinical trial data identified gastrointestinal, skin, and infections as common/uncommon ADRs of DPP4i (**S5**). DPP4i has a well‐established association with these two ADRs (S1)^41^ further confirmed by our study of the Yellow Card Scheme. Our results have confirmed that these are the most reported ADRs and found a minimal statistical difference between the DPP4i drug class.

### 4.1 Pharmacokinetic and physiochemical properties in relation to pharmacological data and ADRs

All five DPP4i were designed around the two basic amino acids; proline and alanine, giving the four DPP4i some structural similarity,^42^ (Figure 1). This in turn generates similar PK and physiochemical properties (Table S2). Notably, linagliptin was the most lipophilic and had the strongest binding interaction with DPP4 and FAP (FAP exhibits the highest sequence identity to DPP4 of the DASH proteins).^37^

Regardless of binding affinity with DPP4, there appears to be no correlation with the reported ADR data.

### 4.2 Gastrointestinal (GI)

One notable ADR across the DPP4i class is acute and chronic pancreatitis with the highest rate associated with linagliptin (2.65 ADRs/1000000 *R*_*x*_) and lowest rate with alogliptin (1.78). Acute pancreatitis in DPP4i therapy warranted an MHRA alert (2014) due to the risk identified via pharmacovigilance.^43^

One possible underlying mechanism is the increased incretin hormone GLP‐1 circulation‐time which induces overgrowth of pancreatic acinar and ductal cells,^44^, ^45^ causing occlusion and resulting in pancreatitis^46^; although it must be noted that β‐cell proliferation was induced with physiologically unlikely concentrations of GLP‐1 and there are conflicting evidence of a significant association of pancreatitis with DPP4i therapy.^47^, ^48^, ^49^, ^50^

DPP8 and DPP9 are more closely associated with gut inflammation and colitis.^51^, ^52^ Saxagliptin has the highest interaction with DPP8 and DPP9 (242 nM and 102 nM, respectively) and exhibits the fewest total GI ADRs and sub‐categorical GI ADRs. This may be attributed to the decreased inflammation associated with DPP8 and DPP9 resulting in less local irritation causing ADRs such as diarrhea, abdominal pains, and nausea and vomiting, for which saxagliptin had the fewest reported side effects across the class. Another suggested mechanism is due to the prolonged anti‐motility activity of GLP‐1.^41^

### 4.3 Skin

DPP4 expression is ubiquitous in the dermis,^53^ and the inhibition of DPP4 contributes to several cutaneous conditions, such as psoriasis,^52^, ^54^ atopic dermatitis,^55^ cutaneous T‐cell lymphoma and keloids,^53^ amongst others.^56^

One rare but notable adverse reaction of DPP4i, is the development of bullous pemphigoid (BP),^41^, ^57^, ^58^ an autoimmune cutaneous disease with uncertain pathogenesis. Histological features of BP vary depending on whether it is DPP4i‐induced or of classical pathogenesis.^53^ DPP4 has an established role in converting plasminogen to plasmin; one suggested mechanism is that DPP4i prevents plasmin cleavage of collagen XVII,^53^, ^59^ resulting in the breakdown of immunotolerance against collagen and the production of autoantibodies against distinct collagen epitopes. Furthermore, enhancement of the activity of proinflammatory chemokines, such as CCL11/exotoxin, results in blister formation,^59^ as well as possible interference with keratinocyte migration and delayed wound healing.^60^

Linagliptin has the highest rate of bullous conditions (4.03) which may be attributed to its moderate affinity with FAP (89 nM); whereas other DPP4i affinity with FAP is significantly weaker. FAP has a defined role in collagen cleavage,^37^ which also promotes macrophage adhesion,^61^ and therefore the inhibition of FAP may lead to an additional autoantibody‐mediated response as well as less macrophage adhesion and activity, which impacts the formation of blisters and skin healing.

### 4.4 Immunological role (joints and infection)

DPP4, also known as CD26, is involved in amplifying the co‐stimulatory signaling required for T‐cell receptor activation and subsequently has an immune modulatory function.^37^ Increased DPP4 activity is associated with decreased severity of rheumatic disease due to the dual role of CD26 involving cytokine inhibition and inducing cellular immunity.^36^ It is logical therefore that DPP4i are associated with increased arthralgia and joint stiffness,^37^ to the extent that an U.S. Food and Drug Administration (FDA) alert (2015) was circulated highlighting the association between DPP4i with severe and disabling joint pain.^62^ Alogliptin was reported to have the highest ADR rates associated with musculoskeletal disorders (6.24) and joint signs and symptoms (3.39) and saxagliptin was reported to have the lowest (1.89 and 0.95, respectively). This could be attributed to the increased affinity of saxagliptin with DPP8/9 that augment the immune modulatory processes of DPP4 and consequently exacerbate rheumatic symptoms.

By suppressing T‐lymphocyte‐activating cytokines, chemokines and peptide hormones, there is an increased risk of infections^41^, ^63^; namely nasopharyngitis, sinusitis, upper respiratory tract infections, and urinary tract infections.^5^, ^64^ Furthermore, the potential inhibitory action of DPP8 and DPP9, which also has known T‐lymphocyte‐activating effects may increase immune modulation and increase the risk of infections.

Saxagliptin had the highest rate of infection (3.79) compared to linagliptin (1.38), alogliptin (1.25), and sitagliptin (0.96), respectively. This may be attributed to the increased affinity for the DPP8, DPP9, and FAP enzymes which, as discussed, all replicate and exaggerate the immunological function of DPP4.^65^ This accumulation of anti‐T‐lymphocyte activation may cause an increased risk of infections; however, we would expect a correlating increase in joint pain or symptoms for saxagliptin if this were the case, although saxagliptin experienced the lowest musculoskeletal ADRs.

### 4.5 Cardiovascular

While alogliptin appears to exhibit a difference in cardiac ADR rate over sitagliptin, statistical significance across the DPP4i class was not reached. An FDA safety review has found that type 2 diabetes medicines containing saxagliptin and alogliptin may increase the risk of heart failure, particularly in patients who already have heart or kidney disease.^66^ These findings were replicated in previous studies investigating the cardiac safety of alogliptin in type II diabetes,^50^, ^67^ which concluded that alogliptin had no additional cardiovascular risk than the placebo. Further trials have conferred that each DPP4i has no additional cardiovascular risk or excessive cardiac toxicity profiles,^47^, ^48^, ^49^, ^50^, ^68^ which is particularly important given that patients with type II diabetes are at two‐to four‐fold increased risk of cardiovascular events.^50^ Furthermore, it has also been postulated that this class exerts cardiovascular protection through GLP‐1 modulation in vivo, which has well‐established cardiovascular effects through coronary artery endothelial cell proliferation and vasculoprotective endothelial progenitor cell stimulation.^69^, ^70^ Acknowledging that DPP4i increase GLP‐1 levels, known benefits of GLP‐1 should theoretically be augmented with DPP4i therapy. Furthermore, DPP4i can reduce pro‐inflammatory cytokines, such as MCP‐1,^71^ which is associated with atherosclerotic plaques and visceral fat, which both contribute to immune‐inflammatory disease, closely associated with diabetes. Reduction in chronic, low‐grade inflammation could result in reduced cardiovascular pathology in type II diabetes. This could explain why the least reported organ class was cardiac disorders and shows DPP4i are associated with minimal cardiovascular toxicity. While there is a theoretical benefit, the major cardiovascular trials for each DPP4i did not confer any cardiovascular benefit in a large‐scale study.^47^, ^48^, ^49^, ^50^, ^68^

### 4.6 Total ADRs

From visual inspection of the ADR rates and IC_50_ inhibition, there is little correlation between selectivity for DPP4, off‐target interactions and total ADRs. Whilst linagliptin had the strongest interaction (1 nM), the total ADR rate for linagliptin was the second highest of all four drugs, suggesting that there is no relationship between strong on‐target interactions and reduced ADRs. Saxagliptin had the lowest ADR rate but only the second‐lowest IC_50_, further indicating little correlation between these factors. This may be attributed to the similar physiological functions across the DPP4 family meaning that on‐target ADRs and other interactions, with varying strengths of interaction, play a role in the overall toxicity profile of a drug.

## 5 LIMITATIONS

Vildagliptin did not fit the scope for data availability and was omitted from the analysis. The Yellow Card Scheme relies on patients and healthcare professionals (HCPs) to report a ‘suspected’ adverse reaction of drug therapy; however, causality does not need to be demonstrated and there is limited information about pre‐existing co‐morbidities, polypharmacy, genetics, or other factors.^10^ Some ADRs may be a result of confounding factors, such as comorbidities, which cannot be identified from the Drug Analysis Profiles. Furthermore, time pressures on HCPs can lead to the under‐reporting of ADRs.^72^

Further factors can influence reporting of ADRs such as media reports or MHRA alerts which may result in increased reports for a period thereafter. The Weber effect is a well‐observed effect where reporting reduces dramatically one‐year post‐approval (black triangle status of new drugs)^73^; considering DPP4i have been licensed for at least 8 years, it is likely that ADRs are now under‐reported. Moreover, patients may stratify side effects in terms of severity and consider some types more concerning than others, leading to higher reporting rates than other ADRs considered less important to report.^17^, ^74^

Post‐marketing surveillance systems also do not quantify the severity of an ADR; for example, one drug might cause a more severe headache than another, but this is not evaluated by the reporting system and therefore cannot be discussed in this study.

The Yellow Card Scheme is a UK‐wide spontaneous reporting system; however, as openprescribing.net is English primary care only, the nationwide ADR rates may appear higher than the true value. OpenPrescribing.net measures the number of items prescribed; however, offers limited information regarding tablet strength or dose prescribed and therefore, the number of items prescribed may not necessarily equal to the number of patients. S4 shows the tablet strengths available for each DPP4i and therefore if multiple tablets are prescribed to make the BNF indicated dose, ADRs may appear higher than the true value (the exception being linagliptin which has only one formulated strength tablet).

Whilst standardization is often ADRs/100000 *R*_*x*_,^19^, ^75^ the resulting numbers were not suitable for Chi‐squared analysis (which is only valid where numbers are >5).^76^ Therefore, standardizing the ADRs per million prescriptions improved the validity; however, it should be noted that any significant difference in the data reported relates to a population‐level risk rather than individual risk.

Although saxagliptin is not licensed as a patch in the UK, the Yellow Card Scheme profile for saxagliptin listed transdermal patch as a formulation. These side effects were included in this study; however, it may lead to a higher rate of skin reactions in comparison to other DPP4i which could not be mitigated against.

## 6 CONCLUSIONS

We have demonstrated that DPP4i have low suspected ADR rates and little statistical difference in types of ADR or differences in pharmacology. Whilst alogliptin appeared to demonstrate more ADRs in most organ classes, which may require further monitoring, this only reached statistical significance in total ADRs and other sub‐categories such as the nervous system (against saxagliptin) and skin (against saxagliptin and sitagliptin).

Saxagliptin, the least prescribed drug, consistently showed the lowest ADR rates in reported classes; again, rarely reaching significance showing the uniformity in ADR profile across the four DPP4i. This study was able to confirm that the most commonly experienced ADRs in this class were the already‐established GI and skin reactions.^41^

We have postulated underlying mechanisms for these ADRs in relation to their pharmacological, physiochemical, and pharmacokinetic profiles.

The results demonstrate that the four DPP4i investigated have very similar structural and pharmacokinetic profiles, except for linagliptin, which showed increased lipophilicity and altered pharmacokinetic activity. Regardless of structural differences, the pharmacological activity against DPP4 did not correlate with the total suspected ADRs.

The non‐DPP4 targets of the DPP4is of possible physiological relevance were identified as DASH proteins and therefore homologs of the desired protein target, meaning most off‐target effects were an augmentation of DPP4‐mediated adverse effects. Namely DPP8, DPP9, and FAP, with their role in immune modulation, or the prolonged action of incretin hormones GIP and GLP‐1.

Further monitoring of pharmacovigilance schemes and a 5‐year review of this data using this methodology may identify the statistical difference between the DPP4is. Continuing monitoring and further research into the cardiac toxicity profile may determine whether there is a protective effect or cardiac damage caused from this therapy.

## NOMENCLATURE OF TARGETS AND LIGANDS

Key protein targets and ligands in this article are hyperlinked to corresponding entries in http://www.guidetopharmacology.org, the common portal for data from the IUPHAR/BPS Guide to PHARMACOLOGY,^77^ and are permanently archived in the Concise Guide to PHARMACOLOGY 2019/20.^78^

## AUTHOR CONTRIBUTIONS

Alan M. Jones and Lauren Jones made substantial contributions to the conception and design of the study; the data were acquired by Lauren Jones; the analysis and interpretation of the data were performed by Lauren Jones and Alan M. Jones; Alan M. Jones and Lauren Jones wrote the draft of the article and revised it critically for intellectual content. The final version was approved by all authors.

## FUNDING INFORMATION

No funding sources to report.

## CONFLICT OF INTEREST

No known or perceived conflicts of interest are disclosed.

## ETHICS STATEMENT

All data collected was publicly available without patient‐identifiable information. No ethical approval or consent was required.

## Supporting information


Appendix S1:

## Data Availability

All underlying data can be found in the supporting materials.

## References

[1] World Health Organisation, Diabetes 2021 10 Nov 2021; Available from: https://www.who.int/news‐room/fact‐sheets/detail/diabetes.

[2] The National Institute for health and care excellence . Type 2 Diabetes in Adults: Management . 2015 24 Nov 2021 21 Dec 2021; Available from: https://www.nice.org.uk/guidance/ng28 26741015

[3] Nauck MA , Meier JJ . Incretin hormones: their role in health and disease. Diabetes Obes Metab. 2018;20(Suppl 1):5‐21. 29364588 10.1111/dom.13129

[4] Ahrén B . Clinical results of treating type 2 diabetic patients with sitagliptin, vildagliptin or saxagliptin – diabetes control and potential adverse events. Incretins. 2009;23(4):487‐498. 10.1016/j.beem.2009.03.003 19748066

[5] Richter B , Bandeira‐Echtler E , Bergerhoff K , Lerch C . Emerging role of dipeptidyl peptidase‐4 inhibitors in the management of type 2 diabetes. Vasc Health Risk Manag. 2008;4(4):753‐768. 19065993 10.2147/vhrm.s1707 PMC2597770

[6] Liu X , Liu Y , Liu H , et al. Dipeptidyl‐peptidase‐IV inhibitors, imigliptin and alogliptin, improve Beta‐cell function in type 2 diabetes. Front Endocrinol. 2021;12:1232. 10.3389/fendo.2021.694390 PMC8488395 34616361

[7] Baetta R , Corsini A . Pharmacology of dipeptidyl Peptidase‐4 inhibitors. Drugs. 2011;71(11):1441‐1467. 21812507 10.2165/11591400-000000000-00000

[8] Shah P , Ardestani A , Dharmadhikari G , et al. The DPP‐4 inhibitor Linagliptin restores β‐cell function and survival in human isolated islets through GLP‐1 stabilization. J Clin Endocrinol Metabol. 2013;98(7):E1163‐E1172. 10.1210/jc.2013-1029 23633194

[9] Sola D , Rossi L , Schianca GP , et al. Sulfonylureas and their use in clinical practice. Archives of Medical Science: AMS. 2015;11(4):840‐848. 26322096 10.5114/aoms.2015.53304 PMC4548036

[10] Gauri S , Can AS , Ricardo C . Pioglitazone. StatPearls Publishing; 2021.

[11] Yoon J‐H , Min SH , Ahn CH , Cho YM , Hahn S . Comparison of non‐insulin antidiabetic agents as an add‐on drug to insulin therapy in type 2 diabetes: a network meta‐analysis. Sci Rep. 2018;8(1):4095. 29511288 10.1038/s41598-018-22443-1 PMC5840350

[12] Pittampalli S , Upadyayula S , Mekala HM , Lippmann S . Risks vs benefits for SGLT2 inhibitor medications. Fed Pract. 2018;35(7):45‐48. PMC6368009 30766374

[13] Garon SL , Pavlos RK , White KD , Brown NJ , Stone CA Jr , Phillips EJ . Pharmacogenomics of off‐target adverse drug reactions. Br J Clin Pharmacol. 2017;83(9):1896‐1911. 28345177 10.1111/bcp.13294 PMC5555876

[14] Wiffen P , Gill M , Edwards J , Moore A . Adverse Drug Reactions in Hospital Patients; a Systematic Review of the Prospective and Retrospective Studies. Bandolier; 2002.

[15] Reddy AS , Zhang S . Polypharmacology: drug discovery for the future. Expert Rev Clin Pharmacol. 2013;6(1):10. 10.1586/ecp.12.74 PMC3809828 23272792

[16] YellowCard . Interactive Drug Analysis Profiles . 22 Dec 2021; Available from: https://yellowcard.mhra.gov.uk/iDAP/.

[17] Huang Y‐L , Moon J , Segal JB . A comparison of active adverse event surveillance systems worldwide. Drug Saf. 2014;37(8):581‐596. 25022829 10.1007/s40264-014-0194-3 PMC4134479

[18] Sandhu D , Antolin AA , Cox AR , Jones AM . Identification of different side effects between PARP inhibitors and their polypharmacological multi‐target rationale. Brit J Clin Pharmacol. 2022;88:742‐752. 34327724 10.1111/bcp.15015

[19] Matharu K , Chana K , Ferro C , Jones AM . Polypharmacology of clinical sodium glucose co‐transport protein 2 inhibitors and relationship to suspected adverse drug reactions. Pharmacol Res Perspect. 2021;9:e00867. 34586753 10.1002/prp2.867 PMC8480305

[20] ChEMBL . Available from: https://www.ebi.ac.uk/chembl/.

[21] National Library of Medicine . PubChem . Available from: https://pubchem.ncbi.nlm.nih.gov/. 10.1080/15360280801989377 28792816

[22] Prasanna S , Doerksen RJ . Topological polar surface area: a useful descriptor in 2D‐QSAR. Curr Med Chem. 2009;16(1):21‐41. 19149561 10.2174/092986709787002817 PMC7549127

[23] Alogliptin . Available from: https://ec.europa.eu/health/documents/community‐register/2015/20150115130399/anx_130399_en.pdf.

[24] Linagliptin . Available from: https://www.ema.europa.eu/en/documents/product‐information/trajenta‐epar‐product‐information_en.pdf.

[25] Saxagliptin . Available from: https://www.ema.europa.eu/en/documents/product‐information/onglyza‐epar‐product‐information_en.pdf.

[26] Sitagliptin . Available from: https://www.ema.europa.eu/en/documents/product‐information/januvia‐epar‐product‐information_en.pdf.

[27] Lobo S . Is there enough focus on lipophilicity in drug discovery? Expert Opin Drug Discovery. 2020;15(3):261‐263. 10.1080/17460441.2020.1691995 31736369

[28] Dudkowski C , Tsai M , Liu J , Zhao Z , Schmidt E , Xie J . The pharmacokinetics and pharmacodynamics of alogliptin in children, adolescents, and adults with type 2 diabetes mellitus. Eur J Clin Pharmacol. 2017;73(3):279‐288. 27999883 10.1007/s00228-016-2175-1 PMC5306220

[29] Graefe‐Mody U , Retlich S , Friedrich C . Clinical pharmacokinetics and pharmacodynamics of linagliptin. Clin Pharmacokinet. 2012;51(7):411‐427. 22568694 10.2165/11630900-000000000-00000

[30] Golightly LK , Drayna CC , McDermott MT . Comparative clinical pharmacokinetics of dipeptidyl peptidase‐4 inhibitors. Clin Pharmacokinet. 2012;51(8):501‐514. 22686547 10.1007/BF03261927

[31] van de Waterbeemd H , Camenisch G , Folkers G , Chretien JR , Raevsky OA . Estimation of blood‐brain barrier crossing of drugs using molecular size and shape, and H‐bonding descriptors. J Drug Target. 1998;6(2):151‐165. 9886238 10.3109/10611869808997889

[32] Gaulton A , Hersey A , Nowotka M , et al. The ChEMBL database in 2017. Nucleic Acids Res. 2017;45(D1):D945‐D954. 27899562 10.1093/nar/gkw1074 PMC5210557

[33] Davies M , Nowotka M , Papadatos G , et al. ChEMBL web services: streamlining access to drug discovery data and utilities. Nucleic Acids Res. 2015;43(W1):W612‐W620. 25883136 10.1093/nar/gkv352 PMC4489243

[34] Mendez D , Gaulton A , Bento AP , et al. ChEMBL: towards direct deposition of bioassay data. Nucleic Acids Res. 2019;47(D1):D930‐D940. 30398643 10.1093/nar/gky1075 PMC6323927

[35] OpenPrescribing.net, EBM DataLab 2022; Available from: openprescribing.net

[36] Busso N , Wagtmann N , Herling C , et al. Circulating CD26 is negatively associated with inflammation in human and experimental arthritis. Am J Pathol. 2005;166(2):433‐442. 15681827 10.1016/S0002-9440(10)62266-3 PMC1602320

[37] Wagner L , Klemann C , Stephan M , von Hörsten S . Unravelling the immunological roles of dipeptidyl peptidase 4 (DPP4) activity and/or structure homologue (DASH) proteins. Clin Exp Immunol. 2016;184(3):265‐283. 26671446 10.1111/cei.12757 PMC4872383

[38] Ross B , Krapp S , Augustin M , et al. Structures and mechanism of dipeptidyl peptidases 8 and 9, important players in cellular homeostasis and cancer. Proc Natl Acad Sci. 2018;115(7):E1437‐E1445. 29382749 10.1073/pnas.1717565115 PMC5816189

[39] Röhrborn D , Wronkowitz N , Eckel J . DPP4 in Diabetes. Front Immunol. 2015;6:386. 26284071 10.3389/fimmu.2015.00386 PMC4515598

[40] Garza A , Park S , Kocz R . Drug Elimination. *Stat Pearls Publishing* ; 2021. 31613442

[41] Huang J , Jia Y , Sun S , Meng L . Adverse event profiles of dipeptidyl peptidase‐4 inhibitors: data mining of the public version of the FDA adverse event reporting system. BMC Pharmacology & Toxicology. 2020;21(1):68. 32938499 10.1186/s40360-020-00447-w PMC7493367

[42] Nojima H , Kanou K , Terashi G , et al. Comprehensive analysis of the Co‐structures of dipeptidyl peptidase IV and its inhibitor. BMC Struct Biol. 2016;16:11. 27491540 10.1186/s12900-016-0062-8 PMC4974693

[43] Agency, M.a.H.p.R. *Dipeptidylpeptidase‐4 inhibitors: risk of acute pancreatitis*. 2014; Available from: https://www.gov.uk/drug‐safety‐update/dipeptidylpeptidase‐4‐inhibitors‐risk‐of‐acute‐pancreatitis.

[44] Perfetti R , Zhou J , Doyle ME , Egan JM . Glucagon‐like Peptide‐1 induces cell proliferation and pancreatic‐duodenum Homeobox‐1 expression and increases endocrine cell mass in the pancreas of old, *Glucose‐Intolerant Rats* . Endocrinology. 2000;141(12):4600‐4605. 11108273 10.1210/endo.141.12.7806

[45] Campbell J , Drucker D . Pharmacology, physiology, and mechanisms of Incretin hormone action. Cell Metab. 2013;17:819‐837. 23684623 10.1016/j.cmet.2013.04.008

[46] Kim Y‐G , Kim S , Han SJ , et al. Dipeptidyl Peptidase‐4 inhibitors and the risk of pancreatitis in patients with type 2 diabetes mellitus: a population‐based cohort study. J Diabetes Res. 2018;2018:5246976. 29850606 10.1155/2018/5246976 PMC5914097

[47] Green JB , Bethel MA , Armstrong PW , et al. Effect of sitagliptin on cardiovascular outcomes in type 2 diabetes. New England Journal of Medicine. 2015;373(3):232‐242. 26052984 10.1056/NEJMoa1501352

[48] Scirica BM , Bhatt DL , Braunwald E , et al. Saxagliptin and cardiovascular outcomes in patients with type 2 diabetes mellitus. New England Journal of Medicine. 2013;369(14):1317‐1326. 23992601 10.1056/NEJMoa1307684

[49] Hanssen NMJ , Jandeleit‐Dahm KAM . Dipeptidyl peptidase‐4 inhibitors and cardiovascular and renal disease in type 2 diabetes: what have we learned from the CARMELINA trial? J Diabetes Complicat. 2019;16(4):303‐309. 10.1177/1479164119842339 PMC6613297 31018682

[50] White WB , Cannon CP , Heller SR , et al. Alogliptin after acute coronary syndrome in patients with type 2 diabetes. N. Engl. J. Med. 2013;369(14):1327‐1335. 23992602 10.1056/NEJMoa1305889

[51] Zhang H , Chen Y , Keane FM , Gorrell MD . Advances in understanding the expression and function of dipeptidyl peptidase 8 and 9. Mol Cancer Res. 2013;11(12):1487‐1496. 24038034 10.1158/1541-7786.MCR-13-0272

[52] Waumans Y , Baerts L , Kehoe K , Lambeir AM , de Meester I . The dipeptidyl peptidase family, prolyl Oligopeptidase and prolyl carboxypeptidase in the immune system and inflammatory disease, including atherosclerosis. Front Immunol. 2015;6:387. 26300881 10.3389/fimmu.2015.00387 PMC4528296

[53] Patel PM , Jones VA , Kridin K , Amber KT . The role of dipeptidyl Peptidase‐4 in cutaneous disease. Exp Dermatol. 2021;30(3):304‐318. 33131073 10.1111/exd.14228

[54] Kridin K , Amber K , Khamaisi M , et al. Is there an association between dipeptidyl peptidase‐4 inhibitors and autoimmune disease? A Population‐Based Study Immunologic Research. 2018;66(3):425‐430. 29855994 10.1007/s12026-018-9005-8

[55] Tasic T , Bäumer W , Schmiedl A , et al. Dipeptidyl peptidase IV (DPP4) deficiency increases Th1‐driven allergic contact dermatitis. Clin Exp Allergy. 2011;41(8):1098‐1107. 21672052 10.1111/j.1365-2222.2011.03778.x

[56] Nakatani K , Kurose T , Hyo T , et al. Drug‐induced generalized skin eruption in a diabetes mellitus patient receiving a dipeptidyl peptidase‐4 inhibitor plus metformin. Diabetes Ther. 2012;3(1):14. 23129260 10.1007/s13300-012-0014-7 PMC3508117

[57] Silverii GA , Dicembrini I , Nreu B , Montereggi C , Mannucci E , Monami M . Bullous pemphigoid and dipeptidyl peptidase‐4 inhibitors: a meta‐analysis of randomized controlled trials. Endocrine. 2020;69(3):504‐507. 32236820 10.1007/s12020-020-02272-x

[58] Yang W , Cai X , Zhang S , Han X , Ji L . Dipeptidyl peptidase‐4 inhibitor treatment and the risk of bullous pemphigoid and skin‐related adverse events: a systematic review and meta‐analysis of randomized controlled trials. Diabetes Metab Res Rev. 2021;37(3):e3391. 32741073 10.1002/dmrr.3391

[59] Chouchane K , di Zenzo G , Pitocco D , Calabrese L , de Simone C . Bullous pemphigoid in diabetic patients treated by gliptins: the other side of the coin. J Transl Med. 2021;19(1):520. 34930319 10.1186/s12967-021-03192-8 PMC8691092

[60] Tasanen K , Varpuluoma O , Nishie W . Dipeptidyl Peptidase‐4 inhibitor‐associated bullous pemphigoid. Front Immunol. 2019;10:1238. 31275298 10.3389/fimmu.2019.01238 PMC6593303

[61] Mazur A , Holthoff E , Vadali S , Kelly T , Post SR . Cleavage of type I collagen by fibroblast activation protein‐α enhances class A scavenger receptor mediated macrophage adhesion. PLOS One. 2016;11(3):e0150287. 26934296 10.1371/journal.pone.0150287 PMC4774960

[62] U.S. Food and Drug Administration . FDA Drug Safety Communication: FDA Warns that DPP‐4 Inhibitors for Type 2 Diabetes May Cause Severe Joint Pain . 2015; Available from: https://www.fda.gov/drugs/drug‐safety‐and‐availability/fda‐drug‐safety‐communication‐fda‐warns‐dpp‐4‐inhibitors‐type‐2‐diabetes‐may‐cause‐severe‐joint‐pain.

[63] Willemen MJ , Mantel‐Teeuwisse AK , Straus SM , Meyboom RH , Egberts TC , Leufkens HG . Use of dipeptidyl peptidase‐4 inhibitors and the reporting of infections: a disproportionality analysis in the World Health Organization VigiBase. Diabetes Care. 2011;34(2):369‐374. 21270195 10.2337/dc10-1771 PMC3024351

[64] Shao S , Xu QQ , Yu X , Pan R , Chen Y . Dipeptidyl peptidase 4 inhibitors and their potential immune modulatory functions. Pharmacol Ther. 2020;209:107503. 32061923 10.1016/j.pharmthera.2020.107503 PMC7102585

[65] Mulvihill EE , Drucker DJ . Pharmacology, physiology, and mechanisms of action of dipeptidyl peptidase‐4 inhibitors. Endocr Rev. 2014;35(6):992‐1019. 25216328 10.1210/er.2014-1035 PMC7108477

[66] FDA Drug Safety Communication: FDA adds warnings about heart failure risk to labels of type 2 diabetes medicines containing saxagliptin and alogliptin. 2018; https://www.fda.gov/drugs/drug‐safety‐and‐availability/fda‐drug‐safety‐communication‐fda‐adds‐warnings‐about‐heart‐failure‐risk‐labels‐type‐2‐diabetes (Accessed 13th April 2022).

[67] White WB , Pratley R , Fleck P , et al. Cardiovascular safety of the dipetidyl peptidase‐4 inhibitor alogliptin in type 2 diabetes mellitus. Diabetes Obes Metab. 2013;15(7):668‐673. 23489301 10.1111/dom.12093

[68] Rosenstock J , Kahn SE , Johansen OE , et al. Effect of Linagliptin vs glimepiride on major adverse cardiovascular outcomes in patients with type 2 diabetes: the CAROLINA randomized clinical trial. JAMA. 2019;322(12):1155‐1166. 31536101 10.1001/jama.2019.13772 PMC6763993

[69] Fadini GP , Avogaro A . Cardiovascular Effects of DPP‐4 Inhibition: Beyond GLP‐1. 2011;55(1):10‐16. 10.1016/j.vph.2011.05.001 21664294

[70] Papagianni M , Tziomalos K . Cardiovascular effects of dipeptidyl peptidase‐4 inhibitors. Hippokratia. 2015;19(3):195‐199. 27418775 PMC4938463

[71] Fadini GP , Boscaro E , Albiero M , et al. The Oral dipeptidyl Peptidase‐4 inhibitor Sitagliptin increases circulating endothelial progenitor cells in patients with type 2 diabetes: possible role of stromal‐derived factor‐1α. Diabetes Care. 2010;33(7):1607‐1609. 20357375 10.2337/dc10-0187 PMC2890368

[72] Molokhia M , Tanna S , Bell D . Improving reporting of adverse drug reactions: systematic review. Clin Epidemiol. 2009;1:75‐92. 20865089 10.2147/clep.s4775 PMC2943157

[73] Beulah E , Reddy N , Subeesh V , Maheswari E , Pudi C . PRM79 ‐ WEBER effect: an extended analysis for ten years of reporting trends IN FDA adverse event reporting system (FAERS). Value Health. 2018;21:S369.

[74] Hazell L , Shakir SAW . Under‐reporting of adverse drug reactions. Drug Saf. 2006;29(5):385‐396. 16689555 10.2165/00002018-200629050-00003

[75] Ferro CJ , Solkhon F , Jalal Z , al‐Hamid AM , Jones AM . Relevance of physicochemical properties and functional pharmacology data to predict the clinical safety profile of direct oral anticoagulants. Pharmacol Res Perspect. 2020;8(3):e00603. 32500654 10.1002/prp2.603 PMC7272392

[76] McHugh ML . The chi‐square test of independence. Biochem Med. 2013;23(2):143‐149. 10.11613/BM.2013.018 PMC3900058 23894860

[77] Harding SD , Sharman JL , Faccenda E , et al. NC‐IUPHAR ; 2018.

[78] The IUPHAR/BPS guide to PHARMACOLOGY in 2019: updates and expansion to encompass the new guide to IMMUNOPHARMACOLOGY. Nucleic Acids Res. 46:D1091‐D1106. doi:10.1093/nar/gkx1121 PMC5753190 29149325

